# Optimal dilation duration of 10 mm diameter balloons after limited endoscopic sphincterotomy for common bile duct stones: a randomized controlled trial

**DOI:** 10.1038/s41598-023-50949-w

**Published:** 2024-01-10

**Authors:** Yuan-Yuan Li, Yin-Shui Miao, Cai-Feng Wang, Jing Yan, Xiao-Jiang Zhou, You-Xiang Chen, Guo-Hua Li, Liang Zhu

**Affiliations:** Department of Gastroenterology, The First Affiliated Hospital, Jiangxi Medical College, Nanchang University, Nanchang, 330006 China

**Keywords:** Gastroenterology, Medical research

## Abstract

Limited endoscopic sphincterotomy (EST) combined with endoscopic papillary balloon dilation (EPBD) is widely used. However, the optimal duration of small balloon dilation in choledocholithiasis remains controversial. We aimed to determine the optimal duration for 10 mm diameter balloon dilation after limited EST in choledocholithiasis. In this randomized controlled clinical trial, 320 patients were randomly assigned to receive small balloon dilation (10 mm in diameter) for 1 min (n = 160) or 3 min (n = 160) after deep bile duct cannulation. No significant difference in success rate of stone extraction between the two groups was observed. The incidence of post-ERCP pancreatitis (PEP) was higher in the 1 min group (10.6%) than in the 3 min group (4.4%) (P = 0.034). The logistic regression analysis showed that guidewire into the pancreatic duct, cannulation time > 5 min and 1 min balloon dilation were independent risk factors for PEP. There were no significant differences in other post-ERCP adverse events such as acute cholangitis, bleeding, perforation, etc. between the two groups. In conclusion, 3 min in duration was determined to be the optimal dilation condition for the removal of common bile duct stones.

## Introduction

In 1974, Classen et al.^1^ and Kawai et al.^2^ introduced endoscopic sphincterotomy (EST), which is effective for the removal of common bile duct (CBD) stones. However, EST has a relatively high risk of bleeding and perforation especially when large EST is performed which is often demanded for stone with diameter > 10 mm and also damages the function of sphincter of Oddi^3^. In 1983, Staritz first used endoscopic papillary balloon dilation (EPBD) for CBD stone removal without compromising sphincter function^4^. As a simpler and safer method than EST, EPBD has fewer negative effects on papillary sphincter function and reduces the probability of bleeding^3^. It is believed that EST plus large-balloon dilation (ES-LBD) is an effect method for treatment of large CBD stones (diameter > 12 mm) because previous studies have confirmed that ES-LBD has similar safety but superior efficiency to conventional treatment (only EST) for the removal of large CBD stones^5,6^. However, ES-LBD is more likely to destroy the function of the sphincter of Oddi and so is neither beneficial nor necessary for the removal of smaller CBD stones (diameter ≤ 10 mm)^7^. In this study, we focused on smaller CBD stones (diameter ≤ 10 mm) because they are more common than larger ones.

Studies had found a relatively high incidence of pancreatitis after EPBD, especially when small (diameter ≤ 10 mm) balloon dilation was performed for small CBD stones (diameter ≤ 10 mm)^8–12^. Interestingly, it has been reported that the incidence of post-EPBD pancreatitis might be related to the duration of balloon dilation^13–16^. Small or limited EST combined with small balloon dilation which is applicable for small CBD stones (diameter ≤ 10 mm) has been reported to have similar efficacy in stone extraction as EST or EPBD alone while preserving partial function of the sphincter, reducing the time of stone removal and minimizing the potential complications^7^. The previous studies of limited EST combine with EPBD varied in the dilation duration^13–16^, but the optimal duration remains unclear. In this study, we explored the optimal dilation duration of EPBD using a small balloon with a diameter of 10 mm after limited EST for CBD stones with diameters ≤ 10 mm.

## Methods

### Patients

**Figure 1:**
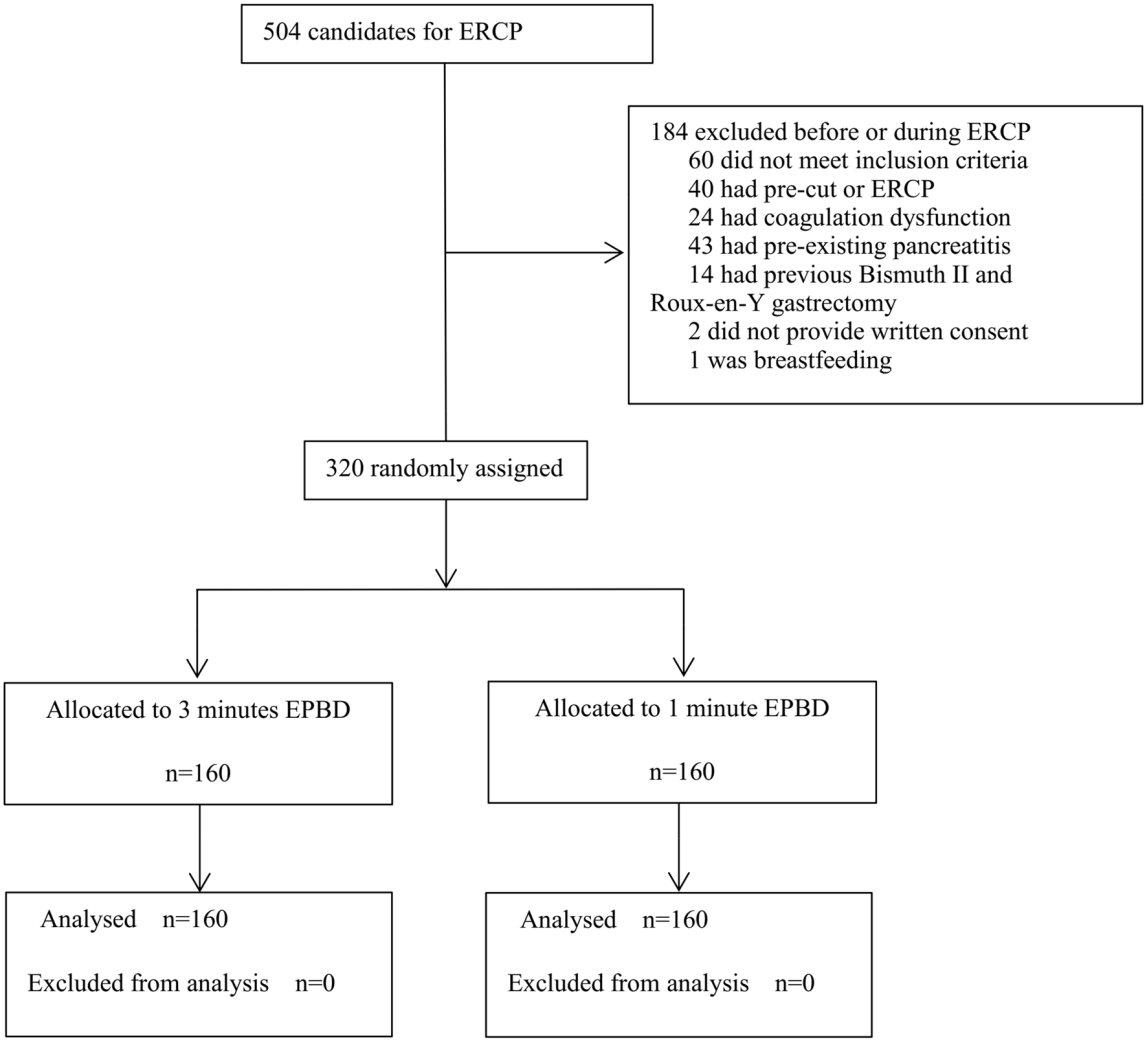
Patient selection diagram.

From January 10, 2018, to December 30, 2019, a single-centre, single-blinded, randomized controlled clinical trial was conducted at the First Affiliated Hospital of Nanchang University. Out of 504 consecutive patients assessed for ERCP, 320 patients fulfilled the inclusion criteria and 184 were excluded before or during ERCP including 60 patients who did not meet inclusion criteria because of inappropriate age or stone diameter (Fig. 1). A computerized random allocation sequence was generated. The statistician combined the random numbers and groups in envelopes and hid their assignments. Specialized nurses enrolled participants, and the doctors assigned participants to interventions. None of these people participated in the data measurement. There were no important changes in the methods and outcomes after trial commencement. There were no harms or unintended effects in any group.

### Eligibility criteria

Inclusion criteria were as follows: (1) age 18–85 years; clinically diagnosed with common bile duct stones (diameter 8–10 mm), and the diameter of the common bile duct was 10–15 mm; (2) no severe cardiopulmonary or renal failure; (3) platelets > 50 × 10^9^/L; (4) PT prolongation less than 3 s; and (5) no usage of anticoagulant or antiplatelet drugs or discontinuation of anticoagulant or antiplatelet drugs for more than 3 days. Exclusion criteria were as follows: (1) patients with pancreatitis; (2) patients for EST pre-cut; (3) patients with a history of EST or EPBD; (4) patients with a long and narrow bile duct, pancreaticobiliary duodenal papillary tumours or biliary cysts; (5) patients with a history of gastrointestinal reconstruction, such as Bismuth II gastrectomy, Roux-en-Y gastrectomy (biliary tube traversed), or pancreaticoduodenectomy; (6) patients with a history of unsuccessful cannulation; (7) patients who refused to participate; and (8) pregnant or breastfeeding patients.

### Ethical consideration

The study was approved by the Institutional Review Board of the First Affiliated Hospital of Nanchang University (No. 2017-056) and registered with the Chinese Clinical Trial Register (Registration date: 02/01/2018, Registration number: ChiCTR1800014259). All methods were performed in accordance with the ethical standards described in the 1964 Declaration of Helsinki and its later amendments.

### Endoscopic procedures

Patients were randomly assigned (1:1) to receive balloon (diameter 10 mm) dilation for 1 min (n = 160) or 3 min (n = 160) after deep bile duct cannulation with a guidewire. The pressure of the balloons was 6 atm. The dilation continued for 1 min or 3 min after the balloon waist disappeared, and the corresponding balloon pressure was reached during cylindrical balloon dilation after limited EST. Limited EST was defined as sphincterotomy performed until the upper margin of the cut portion was located at one third of major EST^17^.

Before the procedure, patients were informed of the conditions and signed informed consent was obtained. Anaesthesia risks were assessed based on each patient’s blood tests, liver function, blood amylase, coagulation functions, electrocardiogram, chest X-ray, ECG (for patients older than 60 years or with a history of heart disease), and lung function or blood gas analysis (for patients older than 60 years or with pulmonary disease). Preoperative magnetic resonance image (MRI) and abdominal B ultrasound were performed to detect common bile duct stones and gallbladder stones. Patients were required to fast for 6–8 h before ERCP. After patients entered the endoscopy room, intravenous anaesthesia with propofol was administered under the close supervision of an anaesthesiologist.

**Figure 2:**
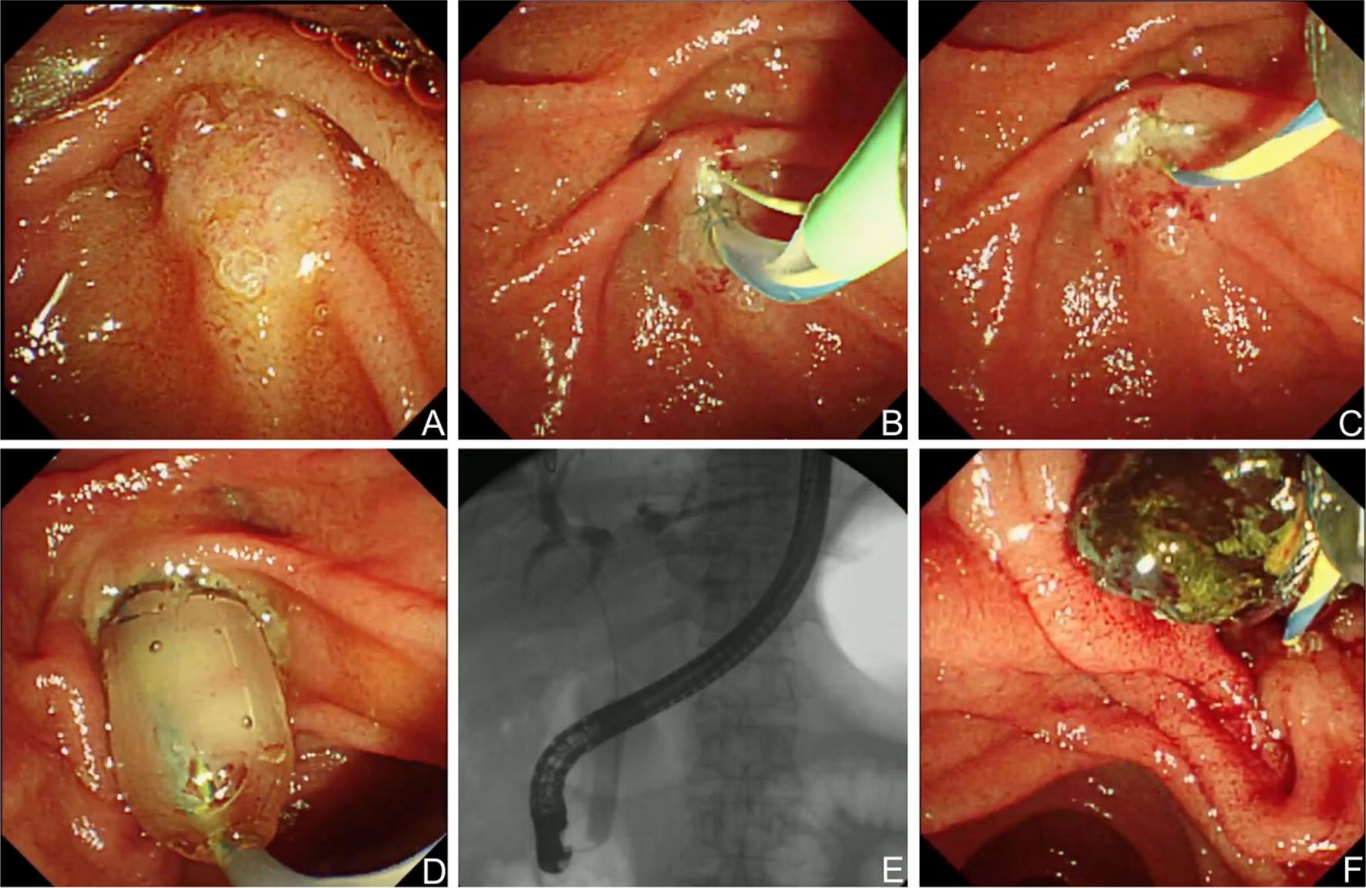
The endoscopic procedure images for EPBD combined with limited EST. (**A**) Intact papilla; (**B**) incision of papilla after successful cannulation; (**C**) Limited EST was performed; (**D**) and (**E**) EPBD (10 mm in diameter) was performed; (**F**) Successful removal of bile duct stones.

The main procedure steps were shown in Fig. 2. The duodenal papilla was found using a duodenoscope (TJF-240; Olympus Optical Corporation, Tokyo, Japan). Then, the sphincterotome (Autotome™ RX 49, Boston Scientific, MA, United States) was withdrawn with a retained guidewire (Hydra Jagwire 0.035 inch, Boston Scientific, MA, United States). The cannulation time started from the contact of the nipple with the sphincterotome until the guidewire successfully entered the bile duct or the operator abandoned the cannulation. After radiography was finished, limited EST was performed. According to the group classification, balloons (Wilson-Cook Medical Inc., Winston-Salem, NC, United States) were inserted to dilate the papillary sphincter. The dilation duration was controlled according to the group classification. The balloon length was confirmed to be longer than the papillary sphincter. A special syringe with a pressure gauge was used to inject a contrast agent into the balloon catheter. The pressure was slowly increased until the balloon waist disappeared and the target pressure was reached. This dilation lasted for 1 min or 3 min. Then, the balloon catheter was removed, and the stones were removed with a basket (WebTM extraction basket, Wilson-Cook Medical Inc., Winston-Salem, NC, United States). When the stone was too large to be removed, mechanical lithotripsy (BML-4Q; Olympus Optical, Tokyo, Japan) was used. Patients who underwent cannulation for more than 5 min or had more than 3 times accidental guidewire insertion into the pancreatic duct were placed with a pancreatic duct stent (Zimmon Pancreatic Stent, Cook Ireland Ltd.) After removing the stones, cholangiography was performed to check whether all stones were removed. If the stone could not be removed during the procedure or after lithotripsy, another ERCP was performed; when there were residual stones, a nasobiliary duct was placed (Nasobil; Sonde, ENDO-FLEX GmbH, Germany) to remove the stones at a later time.

The fasting period lasted 24 h after ERCP. A blood amylase examination was carried out 3 h after ERCP, and routine blood, liver function, and amylase tests were performed after 24 h. Post-ERCP complications, such as fever (oral temperature exceeding 38 °C), abdominal pain, or hemafecia, were evaluated every 8 h after ERCP for at least 48 h. Patients who underwent nasobiliary drainage were treated with cholangiography 2 days after stone removal. If there was no stone residue, the nasobiliary duct was removed. If there were residual stones, stone removal was repeated.

All the endoscopists had more than 5 years of endoscopic experience.

### Evaluation of complications

The primary endpoint was the occurrence of post-ERCP pancreatitis (PEP). Secondary endpoints included the other post-ERCP complications. Diagnostic criteria for PEP were as follows: (1) typical abdominal pain that was severe and unbearable; (2) serum amylase increased to a value over 3 times greater than the upper limit of normal within 24 h after ERCP; and (3) B ultrasound or CT showed changes typical of pancreatitis. PEP was diagnosed by meeting two of the three criteria. The grade of severity of pancreatitis was determined according to the planned fasting period after the ERCP as follows^18^: mild, the extension of the planned fasting period for less than 3 days; moderate, the extension of the planned fasting period for 4 to 10 days; and severe, the extension of the planned fasting period for more than 10 days, surgery or intensive treatment, or death. Other post-ERCP complications, including perforation, bleeding and biliary infection were also monitored in addition to PEP. An abdominal CT examination could show pancreatic morphological changes. The perforation of the digestive tract was represented by sudden abdominal pain and persistent or paroxysmal aggravation accompanied by vomiting and signs of peritonitis. A radiograph or abdominal CT could reveal visible subphrenic free gas. Hemorrhage was represented by melena or hematochezia or the sudden decline of haemoglobin by more than 20 g/L. In each case of haemorrhage, endoscopic haemostasis was required to stop bleeding. Biliary infection referred to an increase in body temperature above 38 °C, with or without pain in the upper abdomen (or right-sided upper abdomen), and an increase in white blood cells and the percentage of neutrophils. Hyperamylasaemia was defined as a condition when only the blood amylase was three times higher than the normal upper limit with no obvious symptoms of abdominal pain.

### Statistical analysis

Based on randomized trials from previous literature^19,20^, we assumed the reported frequency of PEP for the 1 min group would be 20.7%, and it would be 9% for the 3 min group. The theoretical sample size was based on the calculation formula of the comparison of two independent sample rates (α = 0.05, β = 0.2). Thus, 142 patients were needed in each group to detect a difference between groups. Considering a withdrawal rate of 10%, the final estimated sample size per group was 156. Data analysis was performed using SPSS 23.0 (Chicago, IL, United States). All continuous variables were expressed as medians and q25–75 interquartile ranges, and the P values were compared by the Mann–Whitney test. The qualitative variables were analysed by the chi-square test or Fisher’s exact test. Single factor analysis of variance was used to evaluate the relationship between PEP and related risk factors. Statistically significant differences were analysed in the multivariate analysis of independent risk factors for PEP. The multivariate analysis adopted a two-class logistic regression model. P < 0.05 was considered to be statistically significant.

## Results

### General characteristics of patients

There was no significant difference between the EPBD 3 min group and the EPBD 1 min group in patients’ demographic information, stone features, cannulation time, periampullary papillary diverticulum, preoperative cholecystectomy, guidewire into the pancreatic duct, pancreatic duct stent placement or comorbidities (Table 1).

**Table 1:** Baseline characteristics of the patients between the two groups.

	3 min (n = 160)	1 min (n = 160)	P value
Sex (male/female)	87/73	84/76	0.737
Mean age (yr)	61.5 (50.0–72.0)	58.0 (45.3–68.0)	0.067
Stone diameter (mm)	9 (8.4–10)	9 (8.5–10)	0.959
Number of CBD stones			
1	68 (42.5%)	69 (43.1%)	0.910
2	9 (5.6%)	14 (8.8%)	0.279
> 2	83 (51.9%)	77 (48.1%)	0.502
Cannulation time (minute)	1.5 (1.0–2.0)	1.5 (1.0–2.0)	0.412
Preoperative cholecystectomy	43 (26.9%)	45 (28.1%)	0.802
Periampullary diverticulum	58 (36.3%)	46 (28.6%)	0.152
Guidewire insertion into the pancreatic duct	38 (23.8%)	34 (21.3%)	0.531
Pancreatic stent placement	30 (18.8%)	25 (15.7%)	0.459
Comorbidities			
Hypertension	25 (15.6%)	22 (13.8%)	0.636
Heart disease	12 (7.5%)	14 (8.8%)	0.682
Cranial nerve disease	5 (3.1%)	7 (4.4%)	0.556
Diabetes mellitus	16 (10.0%)	22 (13.8%)	0.300
Chronic obstructive pulmonary disease	5 (3.1%)	7 (4.4%)	0.556

CBD, common bile duct.

### Comparison of stone removal success rates and rates of PEP

The success rates of stone removal in the two groups were not significantly different, as stones from 159 patients in the 3 min group (99.4%) and 158 patients in the 1 min group (99.8%) were successfully removed (P = 0.562); stones from 136 patients (85.0%) in the 3 min group and 132 patients (82.5%) in the 1 min group were completely removed in the first session (P = 0.544); and stones from 23 patients (14.4%) in the 3 min group and 26 patients (16.3%) in the 1 min group were completely removed in the second session (P = 0.641). There was no significant difference between the two groups in mechanical lithotripsy (6.9% (11/160) vs. 8.1% (13/160), P = 0.671) (Table 2).

**Table 2:** Comparison of outcomes between the two groups.

	3 min (n = 160)	1 min (n = 160)	P value
Complete stone removal	159 (99.4%)	158 (98.8%)	0.562
Mechanical lithotripsy	11 (6.9%)	13 (8.1%)	0.671
Complete stone removal in 1st session	136 (85.0%)	132 (82.5%)	0.544
Complete stone removal in 2nd session	23 (14.4%)	26 (16.3%)	0.641
Post-ERCP pancreatitis	7 (4.4%)	17 (10.6%)	0.034
Mild	6 (3.8%)	15 (9.4%)	0.042
Moderate	1 (0.6%)	1 (0.6%)	1.000
Severe	0 (0)	1 (0.6%)	0.317
Acute cholangitis	5 (3.1%)	6 (3.8%)	0.759
Perforation	2 (1.3%)	0 (0)	0.156
Bleeding	4 (2.5%)	6 (3.8%)	0.520
Hyperamylasaemia	5 (3.1%)	6 (3.8%)	0.759

The incidence of PEP was higher in the 1 min group (10.6%) than in the 3 min group (4.4%) (P = 0.034). One case of acute pancreatitis in the 1 min group presented with respiratory failure and heart failure; the patient recovered after transfer to the ICU. There was no significant difference between these two groups in the incidence of hyperamylasaemia (3.1% (5/160) vs. 3.8% (6/160), P = 0.759), acute cholangitis (3.1% (5/160) vs. 3.8% (6/160), P = 0.759), haemorrhage (2.5% (4/160) vs. 3.8% (6/160), P = 0.520) and perforation (1.3% (2/160) vs. 0% (0/160), P = 0.156) (Table 2).

### Independent risk factors for PEP

Possible risk factors related to PEP, including age, gender, guidewire into the pancreatic duct, periampullary papillary diverticulum, mechanical lithotripsy, cannulation time > 5 min, and balloon dilation time, were evaluated. Among the above variables, guidewire into the pancreatic duct (OR = 3.499, 95% CI 1.421–8.621, P = 0.006), cannulation time > 5 min (OR = 4.798, 95% CI 1.659–13.872, P = 0.004) and balloon dilation time of 1 min (OR = 3.032, 95% CI 1.148–8.010, P = 0.025) were the risk factors related to PEP (Table 3).

**Table 3:** Logistic regression analysis: prognostic factors for PEP.

Variable	Univariate analysis		Multivariate analysis	
	OR (95% CI)	P value	OR (95% CI)	P value
Gender	1.882 (0.799–4.437)	0.148	2.079 (0.774–5.581)	0.146
Age	0.705 (0.303–1.637)	0.416	0.666 (0.266–1.667)	0.385
Guidewire into the pancreatic duct	3.260 (1.392–7.634)	0.006	3.499 (1.421–8.621)	0.006
Mechanical lithotripsy	2.760 (0.860–8.853)	0.088	2.122 (0.555–8.117)	0.272
Periampullary diverticulum	0.845 (0.339–2.105)	0.717	0.547 (0.192–1.564)	0.261
Cannulation time > 5 min	2.882 (1.196–6.947)	0.018	4.798 (1.659–13.872)	0.004
Balloon dilation time	2.598 (1.047–6.450)	0.040	3.032 (1.148–8.010)	0.025

## Discussion

EPBD has been reported to be comparable to EST in the technical success rate of bile duct stone extraction, but it has a lower risk of bleeding and perforation^3,21^. In addition, EPBD is more effective for the management of difficult stone extraction^21^. However, many studies have reported that EPBD caused a higher incidence of PEP, and the dilation duration might have been associated with PEP and other complications^14,16,19,22,23^. Although most studies used a short dilation time of 1 min or less^16,19,22^, Sato et al.^23^ used longer dilation times (3 repeated 1 min dilations) to fully achieve loosening of the sphincter of Oddi, and no cases of PEP were observed_._ In addition, Bang et al.^14^ found that the incidence of PEP was lower with prolonged dilation (5 min) than with shorter dilation (1 min). Thus, a longer dilation duration seems to decrease the incidence of PEP in the study with EPBD alone. Recent consensus^12^ suggests that, regardless of endoscopic sphincterotomy, dilation with large balloons (diameter 12–20 mm) does not increase the risk of PEP, and the occurrence of PEP is lower than that of small balloon (diameter ≤ 10 mm) dilation. However, large balloons might damage the function of the sphincter of Oddi irreversibly and are not suitable for smaller stones with diameter less than 10 mm^24^.

Currently, the technique of combined limited EST and small balloon dilation has been described as a better alternative method for the exaction of bile duct stones than EST or EPBD alone, because a limited sphincterotomy helps the expansion of the dilation balloon, thus facilitating common bile duct stone extraction. Besides, a small incision causes less bleeding and small balloon dilation reserves partial function of the sphincter of Oddi^7^. However, the optimal duration for the small balloon dilation after limited EST has not been established, with limited related studies. In our study, the incidence of PEP was higher in the 1 min group (10.6%) than in the 3 min group (4.4%) (P = 0.034), which was consistent with the studies on EPBD alone^14,22,23^. A balloon dilation time of 1 min is considered insufficient to relax the intact sphincter of Oddi, resulting in compartment syndrome and oedema, which might increase the occurrence of PEP^12,24^.

According to most of the meta-analyses, the success rates of EST and EPBD for common bile duct stones were 94.3% and 96.5%, respectively^8^. The success rates of combining limited endoscopic sphincterotomy and balloon dilation in our study were 99.4% (3 min group) and 98.8% (1 min group), respectively, which were higher than those reported before, indicating that limited EST combined with balloon dilatation was effective for CBD stone removal. In our study, the mechanical lithotripsy rates were 6.9% (3 min group) and 8.1% (1 min group), respectively. These rates were consistent with the conclusion of Park who reported that combined limited endoscopic sphincterotomy and balloon dilation could reduce the rate of mechanical lithotripsy^25^.

Acute pancreatitis is still the most common early complication of ERCP. In this study, the occurrence of PEP was higher in the 1 min group (10.6%) than in the 3 min group (4.4%) (P = 0.034). It has been reported that the incidence of pancreatitis with EPBD was 4.8–15.1%^26^. Our study indicated that the occurrence of pancreatitis with EST plus EPBD was lower than that with EPBD alone, and EST plus EPBD was a safe and effective method of stone removal. One patient developed severe pancreatitis combined with respiratory failure and cardiac failure after balloon dilation for 1 min but recovered after active treatment. The higher occurrence of PEP observed in the 1 min group could be explained by the fact that the dilation time of 1 min is considered insufficient to relax the Oddi sphincter, resulting in oedema, which could increase the occurrence of PEP^24^. Although two patients had perforations in which the wall of the duodenal descending part was damaged when the endoscope was placed, nylon cord purse-string sutures were used to close the wound and the patients recovered soon after conservative treatment.

A large number of multivariate analyses of risk factors for PEP have suggested that independent risk factors for PEP include patient-related definite risk factors (such as female, patients with a history of pancreatitis, etc.), procedure-related definite risk factors (such as difficult cannulation, etc.), patient-related likely risk factors (such as younger age, etc.) and procedure-related likely risk factors (such as precut sphicterotomy, etc.)^27–32^. In this study, participating patients were rigorously screened so that patients with suspected SOD, previous acute or chronic pancreatitis, nondilated extrahepatic bile duct, end-stage renal disease were excluded. Besides, no enrolled patients in our study had normal serum bilirubin before ERCP. Furthermore, in our study, no pancreatic injection, precut sphincterotomy, pancreatic sphincterotomy or intraductal ultrasound were performed during ERCP. Lastly, the complete stone removal rates of both 3-min group and 1-min group were extremely high (99.4% vs 98.8%). That meant only 1 patient in 3-min group and 2 patients in 1-min group failed to clear bile duct stones, which was not enough for taking failure to clear bile duct stones as a prognostic factor for PEP. So the risk factors mentioned above were not included for analysis. We chose the remaining potential factors including gender, age, guidewire into the pancreatic duct, mechanical lithotripsy, etc. to analyze the prognostic factors for PEP. We found that guidewire into the pancreatic duct (OR = 3.499, 95% CI 1.421–8.621, P = 0.006), cannulation time > 5 min (OR = 4.798, 95% CI 1.659–13.872, P = 0.004) and balloon dilation time of 1 min (OR = 3.032, 95% CI 1.148–8.010, P = 0.025) were the factors related to PEP (Table 3).

It is a fact that several studies^13–16,20^, which have similar study design (exploring the optimal dilation time of endoscopic balloon dilation) with our study have already been reported. However, our studies have differences from them. Most of the previous studies explored the optimal dilation time of EBPD alone^13,14,16^. However, as we mentioned in the introduction part, EPBD alone has a higher risk of PEP than EST^3^. Limited EST combined with EPBD has been reported to have similar efficacy in stone extraction as EST or EPBD while preserving partial function of the sphincter of Oddi, reducing the time of stone removal process and minimizing the potential complications^7^. So it might be more important to determine the optimal dilation time of EPBD followed limited EST because it seems to be better than EST or EPBD alone, but the combination of limited EST and EPBD has been poorly studied. Paspatis et al.^15^ compared a 60-s with a 30-s EPBD after EST, focusing on the large-balloon dilation (diameter > 15 mm) for large bile duct stones (diameter > 12 mm). However, large-balloon dilation is more likely to destroy the function of the sphincter of Oddi and so is neither beneficial nor necessary for the removal of smaller CBD stones (diameter ≤ 10 mm)^7^. As smaller CBD stones (diameter ≤ 10 mm) are more common than larger ones, we focused on CBD stones in smaller sizes in our study, which was different from the study of Paspatis GA et al. Similar to our study, Meng et al.^20^ also determined optimal dilation time for combining small EST and EPBD for CBD stones. However, our result was different from theirs. One of the reasons might be that the balloon sizes were different between the two studies. We analyzed the data of balloons with a diameter of 10 mm, but Meng et al. analyzed the data of balloons with diameters of no more than 12 mm, in which the data of balloons with diameters of 6 mm, 8 mm, 10 mm and 12 mm were combined to compare the effects of different dilation duration on PEP. We believe our result provides an optimal time of 3 min for EPBD with a 10 mm-diameter balloon after limited EST for small CBD stones (8–10 mm in diameter).

Our study had some limitations. First, in our inclusion criteria, only patients having common bile duct stones with a diameter of 8–10 mm and common bile duct with diameter of 10-15mm were included. As we mentioned above, we focused on CBD stones in smaller sizes (diameter ≤ 10 mm) in our study. However, for CBD stones with diameter < 8 mm, limited EST is often enough for stone removal and EPBD is not necessary. So we targeted the CBD stones with the diameter of 8–10 mm because we focused on EPBD after limited EST. Besides, according to the rule of EPBD procedure, the diameter of balloon (10 mm) should not be larger than the diameter of bile duct, so the diameter of bile duct should ≥ 10 mm. On the other hand, an overdilated bile duct is not good for small stone removal so we only targeted patients with common bile duct 10–15 mm in diameter. The relatively narrowly targeted inclusion criteria might affect the popularization of our conclusion. Second, endoscopists in our study were not blinded to the dilation duration, although patients were randomly allocated after successful deep common bile duct cannulation and sphincterotomy. Thirdly, there were only 24 cases of PEP in this study, so the sample size needs to be further expanded to verify the conclusion of multivariate analyses of risk factors for PEP. Finally, this is a single-centre study and more multi-center, randomized controlled clinical trials are needed to obtain a more confirmed conclusion.

In summary, limited EST combined with balloon dilatation is safe and effective for extrahepatic bile duct stones with a high success rate and low incidence of complications. This randomized controlled trial indicated that the efficacy and pancreatitis risk of EPBD is influenced by the dilation duration, and a duration of 3 min is superior to a duration of 1 min.

## Data Availability

The datasets used and/or analyzed during the current study are available from the corresponding author on reasonable request.
